# Electron Beam Irradiation Dose Dependently Damages the *Bacillus* Spore Coat and Spore Membrane

**DOI:** 10.1155/2012/579593

**Published:** 2012-01-26

**Authors:** S. E. Fiester, S. L. Helfinstine, J. C. Redfearn, R. M. Uribe, C. J. Woolverton

**Affiliations:** ^1^Department of Biological Sciences, Kent State University, Kent, OH 44242, USA; ^2^College of Technology, Kent State University, Kent, OH 44242, USA; ^3^College of Public Health, Kent State University, Kent, OH 44242, USA

## Abstract

Effective control of spore-forming bacilli begs suitable physical or chemical methods. While many spore inactivation techniques have been proven effective, electron beam (EB) irradiation has been frequently chosen to eradicate *Bacillus* spores. Despite its widespread use, there are limited data evaluating the effects of EB irradiation on *Bacillus* spores. To study this, *B. atrophaeus* spores were purified, suspended in sterile, distilled water, and irradiated with EB (up to 20 kGy). Irradiated spores were found (1) to contain structural damage as observed by electron microscopy, (2) to have spilled cytoplasmic contents as measured by spectroscopy, (3) to have reduced membrane integrity as determined by fluorescence cytometry, and (4) to have fragmented genomic DNA as measured by gel electrophoresis, all in a dose-dependent manner. Additionally, cytometry data reveal decreased spore size, increased surface alterations, and increased uptake of propidium iodide, with increasing EB dose, suggesting spore coat alterations with membrane damage, prior to loss of spore viability. The present study suggests that EB irradiation of spores in water results in substantial structural damage of the spore coat and inner membrane, and that, along with DNA fragmentation, results in dose-dependent spore inactivation.

## 1. Introduction

*Bacillus *species are notable agents of human infectious disease. The spores of *B. cereus* and *B. subtilis* are especially known as food contaminants, while *B*. *anthracis *is now infamous for its use as a bioterror agent [1]. *B*. *anthracis *was identified in the late 1800s by Robert Koch as the causative agent of anthrax. Anthrax is a disease of antiquity whose characteristic pathology is well recognized and speculated to be the fifth and sixth plagues of the Bible, as well as, the “black bone” disease that ravaged Europe in the 1600s [2]. More recently, *B. anthracis *has come to the forefront of public awareness due to the 2001 deliberate release of its spores through the US postal system, resulting in the death of five people and the sickening of dozens more [3]. Anthrax, like other diseases caused by members of the genus *Bacillus*, is problematic in its vegetative form [4]. Its spore form is induced as a survival stage when environmental conditions deteriorate.

Control of *Bacillus* disease begs a technique, chemical, or technology that effectively kills the vegetative bacteria and prevents spore outgrowth. Antibiotic chemotherapy is used to control human infection. Chlorine dioxide and vaporized hydrogen peroxide are used for decontamination of large contaminated spaces, such as the US postal facilities, congressional offices, and other sites contaminated in 2001 [5]. Electron beam irradiation (EBI) was used to sterilize contaminated mail [6]. EBI was chosen to decontaminate the mostly paper-based mail because of its recognized effectiveness in sterilization of medical devices [7] and foods [8], its short processing time, its use of a nonradioactive energy source, and its high throughput capability [9]. Ironically, there is a paucity of data reporting the direct effects of EBI on bacterial spores, even though it is widely used. Importantly, clonogenicity data have identified D_10_ values of 1–4 kGy for *Bacillus* spores in aqueous environments treated by EBI [10, 11]. These D_10_ values are similar to D_10_ values obtained when *Bacillus* spores are irradiated by radioactive sources [12, 13].

In general, ionizing radiation is well known for causing cellular damage, both by direct effects on biomolecules and indirectly by generating reactive oxygen species that oxidize biomolecules [14–17]. It seems that the early data linking cytotoxicity, induced by ionizing radiation, with DNA damage stifled the search for other potential mechanisms by which ionizing radiation acts on *Bacillus* spores [18]. We have evaluated the impact of EBI on the spore structure using techniques that address membrane integrity changes independent of DNA damage.

Bacterial endospores are dormant cells whose production is stimulated by starvation and whose purpose is survival of the cellular genome [19]. The endospore itself is composed of an innermost core covered (sequentially) by an inner forespore membrane, cortex, outer forespore membrane, and spore coat [20] surrounding supercoiled DNA. The spore coat consists of approximately 30 spore-specific proteins [21] that assist the spore with its survival properties [22]. The spore coat helps to confer resistance against heat (120°C, 15 min), lysozyme, chemical disinfection (0.05%, sodium hypochlorite at 30 min; 500 mg L^−1^ ethylene oxide at 30 min; or 0.88 mol L^−1^ hydrogen peroxide), and low-dose (<10 kGy) gamma irradiation [23, 24]. While the precise function of the outer membrane (a structure essential in spore formation) is unknown, the functions of the cortex and the inner membrane have been defined [25]. Together, the spore coat and inner membrane provide direct resistance to DNA damage by excluding harmful chemicals from the core. The cortex, composed of peptidoglycan, facilitates water reduction from the core [26], and the inner membrane provides a strong permeability barrier against chemicals that may harm the chromosomal DNA within the core [27]. In addition to DNA, the core also contains a large amount of pyridine-2,6-dicarboxylic acid (dipicolinic acid [DPA]) complexed with calcium ions, acid-soluble spore proteins (SASPs) that protect nucleotides, enzymes, ribosomes, various tRNAs, and minimal amounts of water [4, 26, 28]. The large amount of DPA reduces core water content and substantially alters the UV photochemistry of the spore DNA; in fact, it is the combination of these properties that confers resistance to specific forms of radiation [26]. The reduced water content of the core may make it difficult for ionizing radiation to generate DNA-damaging free radicals. Yet, sufficient free radicals are produced to kill spores irradiated by these sources. DPA and SASPs are associated with UV radiation resistance [26] and may also have a role in resistance to other forms of radiation. The aforementioned properties make *Bacillus* spores extremely difficult to eradicate.

*B. anthracis* is listed by the Centers for Disease Control and Prevention as a Category A select agent, classifying it as a substantial public health threat. Thus, other less pathogenic members of *Bacillus* are used as surrogates in the study of *B. anthracis*. Historically, *B. atrophaeus *has been used as an anthrax surrogate because of its low pathogenicity and unique colonial characteristics, even though *B. atrophaeus *is more closely related to* B. subtilis *and *B. anthracis *is most closely related to *B. cereus* [29, 30]. *B. atrophaeus *has been used to study many inactivation techniques, such as gamma irradiation, EB irradiation, and chemical sterilization [9, 30, 31]. Here we report the dose-dependent effects on the structural integrity of the spore inner membrane, coat, and DNA of EB-irradiated *B. atrophaeus* spores.

## 2. Experimental

### 2.1. EB Irradiation of Spores

*B. atrophaeus* (American Type Culture Collection [ATCC] 9372) spores were grown, isolated, and washed free of exogenous cellular debris as described by Helfinstine et al. [9]. Briefly, *B. atrophaeus* was grown on trypticase soy agar supplemented with 5% sheep's blood (Becton Dickinson, Sparks, MD), aseptically harvested, and passed onto nutrient sporulation agar. Bacteria were grown on sporulation agar for 48 h at 36°C followed by growth at room temperature until plates contained greater than 90% spores. Spores were collected by washing the agar surface with 4°C sterile, distilled water (DW) and centrifuging at 2504 × g for 10 min at 25°C (Centra MP4R, IEC, Needham Heights, MA, USA). The spore pellet was washed in phosphate-buffered saline (pH 7.2) supplemented with 0.05% Tween 20 (Mallinckrodt Baker, Inc., Paris, KY) for 5 min. The spore suspension was subsequently washed four additional times with DW to remove tween. The final spore suspension was adjusted to 10^9^ CFU cm^−3^ in DW.

 Electron beam irradiations were conducted with a 5.0 MeV Dynamitron electron beam accelerator (Radiation Dynamics, Inc., Edgewood, NY). Absorbed dose measurements were performed using a liquid radiochromic dosimeter, PRC solution (Far West Technology, Inc., Goleta, CA, USA), to determine the parameters (cart speed and beam energy) required to calculate final EB doses. To allow for maximum penetration of electrons through matter, the EB accelerator was operated at its maximum beam energy (5.0 MeV) with a beam current of 10 mA, resulting in a total power of 50 kW.

Spore suspensions or liquid dosimeters were housed in low-density polyethylene bags (Whirl-Paks, 118 mL, 7.5 × 18.5 cm, <1 mm thick, NASCO, Fort Atkinson, WI) that were subsequently secured to a polystyrene platform on a cart conveyor system (SI Handling Systems, Easton, PA). The cart was ferried under the electron beam with EB doses determined by cart speed. Spore suspensions (*n* = 3 per dose) were exposed to EB doses ranging from approximately 0 to 20 kGy. Irradiated spores were aseptically transferred to sterile 50 mL tubes and held at 4°C until evaluated together.

### 2.2. Dosimetry

**Figure 1:**
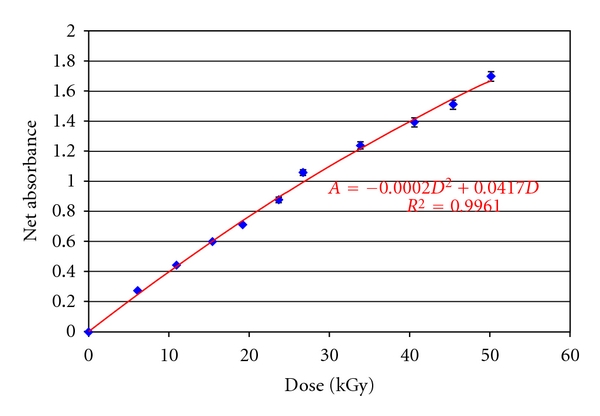
Dose-response curve for a liquid radiochromic dye solution calibrated with alanine pellets as described in the experimental section of this work. Trendline regression was obtained with *r* ^2^ = 0.9961 and a maximum uncertainty of 1.52% with a coverage factor of 2.

Irradiation conditions to give the samples the absorbed doses mentioned above were determined by dosimetry using a radiochromic dye solution (FWT-70-127). This solution was calibrated with alanine pellets to determine the dose-response curve of the solution [32]. Briefly, five mL of the dye solution was poured into the same type of plastic bags used to hold spore suspensions, and five alanine pellets were placed in additional plastic bags. The bags containing alanine pellets and bags with the liquid radiochormic dye solution (PRC) were placed in a Styrofoam Phantom (GEX Corporation) and irradiated using different speeds of the cart conveyor system to control dosing. The alanine pellets had been previously calibrated against alanine films used as a transfer standard dosimeter. After irradiation, the dose was determined from the pellets by measuring the concentration of free radicals induced in them by the irradiation, using a Bruker eScan spectrometer. The absorbance was determined from one mL of the PRC solution (in 1 mm path cuvettes) evaluated at 554 nm in a UV-Vis Perkin Elmer spectrophotometer, model lambda 18 (Perkin Elmer Life and Analytical Sciences, Inc, Boston MA, USA). Dosimetry measurements were repeated five times each, on two separate days (*n* = 10 replicates per dose). Using a 95% confidence limit, the percent error for doses 5, 10, 15, and 20 kGy was 0.7, 0.8, 0.4, and 0.4, respectively. This allowed for the construction of the dose-response curve (Figure 1) that was used to determine irradiation dose when the spore samples were irradiated.

Since dose was a function of the cart speed, the above procedure also determined the cart rate needed to achieve a particular dose. These data (a table of dose versus cart speed, not shown) were used along with (1) (below) to calculate the process constant “*k*,”

(1) $$\begin{array}{c} D=\frac{\left(k\times I\right)}{v}, \end{array}$$

where *D* is the dose (kGy), *I* is the current (10 mA), and *v* is the cart speed (cm/s). In this case, the value of *k* was 11.0 kGy-cm/mA-s. Thus, cart speeds determined from (1) were used to estimate irradiation dose. Spore samples were irradiated along with PRC solution dosimeters, and the dose was measured after each experiment from the PRC dose-response curve, to determine the exact dose provided to the samples. Dose uniformity to the samples was achieved by irradiating the samples with a 100% scanning width of the accelerator, whose uniformity was previously determined [9], and by assuring that the thickness of the irradiated sample was much smaller than the range of 5.0 MeV electrons in water (0.2 cm sample thickness versus 2.4 cm range of electrons).

### 2.3. Spore Clonogenicity

Spore reproduction was determined by use of the standard plate count technique. Briefly, one mL serial dilutions (1 : 10) of irradiated spores or their respective controls were mixed into molten nutrient agar and incubated at 37°C for 48 h. The resulting bacterial colonies arising from individual, germinated spores were counted and reported as the geometric mean of bacterial counts (±standard deviation (SD)) for each EB dose.

### 2.4. Evaluation of Spore Content Loss

Supernatants from washed and standardized spore suspensions irradiated by EB were evaluated for release of spore contents as measured by absorbance at 260 nm, using a NanoDrop ND-1000 spectrophotometer (NanoDrop Technologies, Wilmington, DE, USA). Replicate samples were measured and reported as the geometric mean ± SD.

### 2.5. Impact of EB Irradiation on Spore Genomic DNA

Genomic DNA was isolated from irradiated spores or controls using an UltraClean Microbial DNA Isolation Kit (MO BIO Laboratories, Inc, Carlsbad, CA, USA) according to the manufacturer's instructions, with modification. Briefly, irradiated and control spores were frozen at −80°C for five minutes and rapidly heated to 65°C for two minutes to facilitate spore lysis and release of nucleic acid. Spore genomic DNA was isolated after RNAse treatment and measured by an Eppendorf BioPhotometer (Eppendorf, Hamburg, Germany), to standardize DNA samples for electrophoresis. DNA was stored at −20°C in Tris-acetate buffer (without EDTA), pH 7.2 (Fisher Scientific, Pittsburgh, PA, USA) prior to use until all samples could be evaluated together.

Genomic DNA from spores exposed to EB was analyzed by agarose (0.75%) gel electrophoresis using TAE buffer (40 mmol l^−1^ Tris-acetate, 1 mmol l^−1^ EDTA, pH 8.0) and a 1 Kb Plus DNA ladder (Invitrogen, Carlsbad, CA, USA) to standardize DNA size. Standardized DNA samples from each irradiation dose were loaded with SYBR Gold nucleic acid gel stain (Molecular Probes, Eugene, OR, USA). Electrophoresis was conducted at 100 V for 90 min. DNA was visualized with the Gel Doc reporting system (Bio-Rad Laboratories, Hercules, CA) and DNA concentration quantified using the Quantity One software program (Bio-Rad Laboratories).

### 2.6. Determination of Spore Coat and Membrane Integrity

Irradiated spore samples were also evaluated for spore coat and membrane integrity using vital dyes. Spores (eight log_10_ mL^−1^) were stimulated to germinate in Luria Broth (Becton Dickson Co. Sparks, MD, USA) by the addition of 10 mmol l^−1^ L-alanine (Acros Organics, Geel, Belgium) and the incubation for one hour at 37°C. Germinating spores were then collected by centrifugation (2504 × g for 10 min at 25°C, IEC Centra MP4R, Needham Heights, MA), resuspended in filter-sterilized distilled water (DW), and stained using the LIVE/DEAD BacLight (Molecular Probes, Eugene, OR, USA) bacterial viability method for 15 min at room temperature (Molecular Probes product information sheet, revised 7/15/04). As a positive control, nonirradiated spores were exposed for one hour to 50% hypochlorous acid and then stained according to the LIVE/DEAD BacLight method. Spores were diluted to seven log_ 10_ mL^−1^ in DW and evaluated using a BD FACSAria flow cytometer (BD Biosciences, San Jose, CA, USA). Fifty thousand cells from each sample were analyzed. SYTO 9 signals were collected by a 515–545 nm filter, with the photomultiplier voltage at 610 V; propidium iodide (PI) signals were collected by a 600–620 nm filter at 714 V. Forward and side scatter data were also collected. Data were analyzed using the BD FACSDiva software (BD Biosciences). Changes in the total parent populations of gated quadrants on scatter plots of PI versus SYTO 9 were monitored to determine dye uptake and evaluate spore viability. Forward scatter (FSC) and side scatter (SSC) data were analyzed to report spore size and topology, respectively.

### 2.7. Visualization of Spore Coat Using Electron Microscopy

Irradiated spore suspensions were centrifuged at 2504 × g for 10 min at 25°C (IEC Centra MP4R, Needham Heights, MA, USA) to obtain spore pellets. Supernatants were saved for analysis of irradiation-released spore content, as above. Pellets were dried overnight under a sterile airstream. Once dried, the spore pellets were adhered to aluminum stubs for visualization by electron microscopy. Spore samples were sputter coated with gold in an Anatech LTD sputter coater (model Hummer VI A, Alexandria, VA, USA) at a current of 15 mA for 1.5 min and examined using a JEOL JSM-35C (Japan Electron Optics Laboratory Ltd., Tokyo) scanning electron microscope at 25 kV.

### 2.8. Statistical Analysis

 A linear regression line from the plot of EB dose versus log_10_ CFU mL^−1^ inactivation was used to predict the EB dose required to reduce the number of viable *B. atrophaeus* spores by 90% (D_10 _value). Spectroscopy and cytometry data were analyzed by one-way analysis of variance (ANOVA) using the GraphPad Instat software (GraphPad Software, Inc., San Diego, CA, USA). The Tukey-Kramer multiple comparisons posthoc test was used to compare the mean of the control group (nonirradiated spores) with the means of irradiated spore groups. Significance was set *a priori* at *P* ≤ 0.05.

## 3. Results

### 3.1. Effect of EB Irradiation on Clonogenic Activity

**Figure 2:**
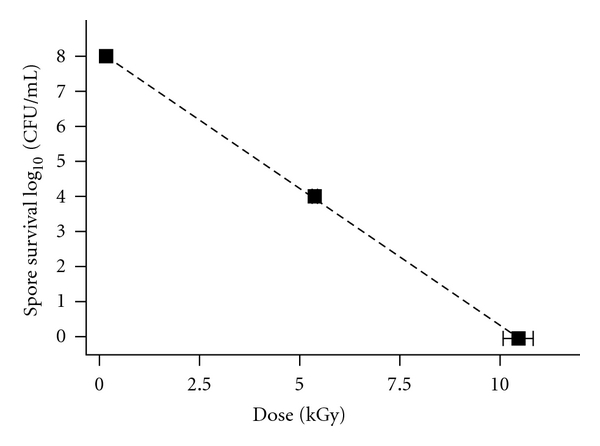
Inactivation of *B. atrophaeus* spores as a function of electron beam irradiation dose (*n* = 3). Linear regression (*r* ^2^ = 0.999) extrapolation produced a D_10_ value of 1.3 kGy. Error bars in both the *X*- and *Y*-axis directions indicate the accuracy in the measurements of dose and colony counts, respectively.

An EB dose of 5.3 ± 0.3 kGy resulted in a four-log reduction of spore viability as determined by standard plate counts; a total loss of spore reproduction occurred at an EB dose of 10.4 ± 0.7 kGy (through an average of 8.0 mm of water). A regression line (*r* ^2^ = 0.999) was used to extrapolate a D_10 _value of 1.3 ± 0.1 kGy (Figure 2). (Previous studies indicated that the highest EB dose (21.7 kGy) only increased the temperature of spore suspensions to 40°C, well below the lethal temperature for *B. atrophaeus* [9, 33].)

### 3.2. Effect of EB on Release of Spore Cytoplasmic Content

The loss of spore cytoplasmic content was found to be correlated with EB dose. Supernatants from irradiated spores were evaluated for spore cytoplasmic contents at 260 nm. Materials that absorbed at 260 nm were released proportionally to absorbed EB dose. The concentrations of these materials were 0.57 ± 0.26 ng *μ*L^−1^ at 0 kGy (nonirradiated), 2.02 ± 0.17 ng *μ*L^−1^ at 5.3 kGy, 3.07 ± 0.33 ng *μ*L^−1^ at 10.4 kGy, 3.18 ± 0.39 ng *μ*L^−1^ at 16.0 kGy, and 33.17 ng *μ*L^−1^ ± 2.25 ng *μ*L^−1^ at 21.7 kGy. Irradiation at 5.3 kGy did not produce a statistically significant difference from the unirradiated control; all other irradiations resulted in statistically significant differences (*P* < 0.01) from the unirradiated control by the Tukey-Kramer test.

### 3.3. Effects of EB Irradiation on Genomic Spore DNA

**Figure 3:**
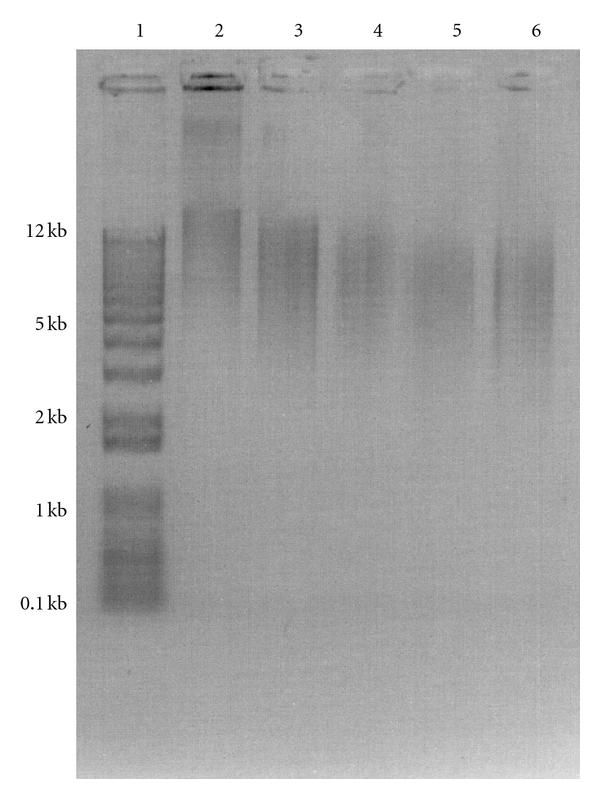
Representative agarose gel used in densitometric analysis of DNA recovery from spores irradiated with varying doses of EB radiation: lane 1, 1 kB Plus DNA Ladder; lane 2, nonirradiated spore DNA; lane 3, DNA from spores irradiated with 5.3 kGy; lane 4, DNA from spores irradiated with 10.4 kGy; lane 5, DNA from spores irradiated with 16.0 kGy; lane 6, DNA from spores irradiated with 21.7 kGy.

Genomic DNA extracted from irradiated spores was measured to determine the effect of EB dose on DNA integrity. The amount of genomic DNA recovered from irradiated spores was 1.616 *μ*g, 1.609 *μ*g, 1.473 *μ*g, 1.387 *μ*g, and 1.324 *μ*g at 0 kGy, 5.3 kGy, 10.4 kGy, 16.0 kGy, and 21.7 kGy, respectively. DNA concentrations were standardized to 1.6 *μ*g per well prior to electrophoresis. Gel electrophoresis also demonstrated genomic DNA degradation as a function of increasing EB dose (Figure 3).

### 3.4. EB Effects on Spore Coat and Membrane Integrity

**Figure 4:**
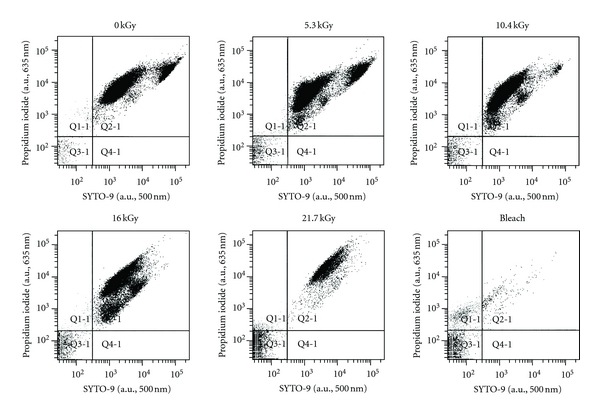
Representative FACS scatter plots of *B. atrophaeus* spores stained with propidium iodide and SYTO 9. Spores were exposed to various doses of EB irradiation (listed above respective scatter plots) or 50% hypochlorous acid prior to assessment of fluorescent dye uptake.

Increasing EB dose resulted in decreasing inner spore membrane and coat integrity as measured by LIVE/DEAD fluorescence (Table 1). Comparison of unirradiated spores with those that were EB-irradiated demonstrated that germination was not induced by EB irradiation, but by L-alanine (data not shown). EB irradiated spores, induced to germinate with L-alanine, took up both SYTO 9 and propidium iodide (PI). Viable spores are known to take up both SYTO 9 and PI upon germination presumably by water influx events [34]. Spores with intact inner spore membranes and coats (strongly SYTO 9-positive) were distinguished from spores with compromised membranes and coats (strongly PI-positive) by comparison with nonirradiated controls and hypochlorous acid-treated spore samples. The total spore population was segregated into four subpopulations (quadrants) so as to monitor EB irradiation effects on dye uptake (Figure 4). An increase in EB irradiation decreased the number of SYTO 9-positive spores (i.e., spores in quadrant 2) and increased the number of strongly PI-positive spores (in quadrant 3), in a dose-dependent manner (Table 1). Furthermore, the higher EB doses resulted in the substantial destruction of spores, resulting in less than the required 50,000 spores to be evaluated (Figure 4). The ratio of SYTO 9-fluorescence to PI-fluorescence in quadrant 2 indicates a significant decrease in the viable spore population, resulting from an increasing EB dose (Table 2). Additionally, increasing the EB dose resulted in decreased forward scatter (spore size) of spores with a concomitant increase in side scatter, altered spore topology (Table 3).

### 3.5. EB Induced-Spore Coat Damage Visualized by Electron Microscopy

**Figure 5:**
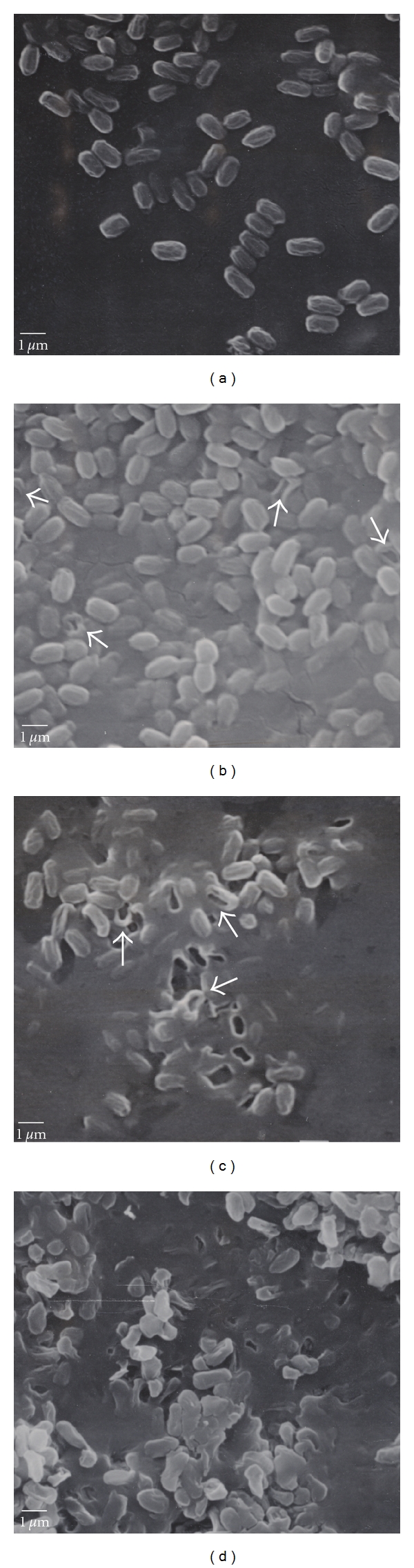
Scanning electron micrographs illustrating spore damage at varying EB dosages: (a) control spores (5,400 x magnification), (b) 5.3 kGy (5,400 x), (c) 10.4 kGy (5,400 x), and (d) 21.7 kGy (5,400 x). Scale bar is 1.0 *μ*m. White arrows indicate ruptured spores.

In Figure 5, scanning electron micrographs of spores exposed to EB irradiation illustrates a dose-dependent increase in spore coat damage: viable, undamaged spores, 0 kGy (Figure 5(a)); visible holes (arrow) in the spore coat of an occasional spore, 5.3 kGy (Figure 5(b)); prominent damage (arrows) to the spore coat, 10.4 kGy (Figure 5(c)) and 21.7 kGy (Figure 5(d)) with apparent cytoplasm leakage (Figure 5(d)).

## 4. Discussion

EBI is an effective method for the decontamination of devices, food, and contaminated mail, effectively inactivating bacterial spores [9, 35, 36]. EBI decreases bacterial spore viability in both dry and liquid environments, as evidenced by very similar D_10_ values [9, 35, 36]. EBI results in the direct chemical alteration of atoms resulting from energy capture [15, 16, 31] and the expulsion of electrons from atomic orbitals, altering electrically neutral atoms or molecules into charged free radicals [8, 37]. Damage caused by radiation-induced radical formation is affected primarily by spore water content and the presence of oxygen, the latter being required for maximal reduction in spore viability [38, 39]. Interestingly, spore radiation sensitivity is reduced at very low water content (such as at vapor pressures approaching ambient air); yet, radiation sensitivity is increased in spores suspended in water [39]. This sensitizing effect is due to the production of radiolytic products of water in the presence of oxygen—in particular, hydrogen peroxide and hydroxyl radicals [40]. While the extremely low water content in spore cores should substantially mitigate the effects of ionizing radiation-induced free radicals, sufficient water appears to remain to permit free radical damage from EBI [26]. Furthermore, free radicals from environmental materials adjacent to spores can result in spore damage during EBI, as well. Data from this investigation demonstrate that EBI results in substantial structural damage of water-borne spores, that is, loss of spore coat integrity and DNA degradation, in addition to other events leading to radiation-induced spore killing. Physical damage to the spore coat caused by EB-induced free radicals, especially at EB doses insufficient to substantially degrade genomic DNA, results in substantial loss of viable spores.

While some of the spore damage can be attributable to EB-induced free radicals from water, structural damage to the spore membrane and DNA likely results from EB-induced events within the spore. In addition to direct visualization of EB-induced spore damage and leakage, EB irradiation dose dependently increased the measurable release of cytoplasmic materials, increased uptake of PI (an indicator of spore membrane integrity loss), decreased SYTO 9 fluorescence (an indication of declining cell viability), and altered spore size and topology. In other words, it appears that EBI acts on water-borne spores from both inside and outside of the spore.

 Our calculated D_10_ was 1.3 ± 0.1 kGy in this water-based spore-killing system, which is similar to values previously published [11, 41]. A four-log reduction in the number of viable spores occurred with an EB dose of 5.3 kGy, correlating with altered spore size and shape, increasi ng spore membrane damage measured by dye uptake and the appearance of occasional spore coat damage visualized by electron microscopy (arrows in Figure 5(b)). An eight-log reduction of spores (loss of total spore viability), marked genomic DNA degradation, cytoplasm leakage, and pronounced damage of spore coats resulted from an EB dose of 10.4 kGy. De Lara et al. [10] also noted the need for at least 10 kGy of EBI to inactivate spores (in foodstuffs). The altered spore surface features we report indicate that free radicals derived from the water vehicle (H atoms and OH radicals, according to Tallentire and Powers [39]) likely attacked the spores from the outside. Damage leading to membrane failure and the resulting DNA degradation at the lower EB doses appear to have resulted from EB-induced free radicals within the spores, although this is yet to be determined. Spore damage was exacerbated at EB doses of 16.0 and 21.7 kGy, validating the substantial damage EBI can cause to spores held in aqueous environments.

It is important to note that spore sterilization can be due to loss of DNA integrity, the loss of spore coat integrity, or both. Evidence of dose-dependent EBI spore coat damage is reported by flow cytometry measuring increasing PI uptake and the simultaneous decrease in SYTO 9 uptake. SYTO 9 generally labels all cells, while PI only penetrates cells with damaged membranes. Nonirradiated spores were primarily undamaged, stained well with SYTO 9, demonstrated characteristic *Bacillus* spore size and shape, and were thus used as a negative control. SYTO 9 and PI fluorescence values were used to construct a ratio (SYTO 9/PI) to evaluate EB-induced changes. Increasing PI fluorescence, with decreasing SYTO 9 fluorescence, clearly predicts declining spore integrity as a function of increasing EBI. Our results do not reveal the timing or sequence of events resulting in spore inactivation. Nonetheless, the data of this study report an EB dose effect where structural damage to spore coats and/or DNA occurring at 5.3 kGy is sufficient to inactivate 4 logs (99.99%) of the spores, which then progresses to spore disintegration at 21.7 kGy, with the loss of total spore viability (8 logs) near 10.4 kGy.

EBI is very effective for the decontamination of *Bacillus* spores. DNA damage induced by ionizing radiation [14–17] has been previously suggested as the mechanism by which EBI inactivates *Bacillus* spores. We also confirm EBI-induced degradation of DNA. However, our data also indicate that EBI damages the *Bacillus* spore coat and the forespore membrane, in addition to degrading genomic DNA, in a dose-dependent manner. Thus, we conclude that the increasing spore coat and membrane damage of the EB-irradiated spore plays an important role in decreasing spore viability. Clearly, spore coat damage, altered membrane permeability, and subsequent spore leakage play a significant role along with DNA fragmentation to result in bacterial spore inactivation. While determining the exact sequence of EBI-induced damage to spores was not the goal of this study, identification of spore coat and membrane damage can assist in tuning EBI to control spore contamination.

## Figures and Tables

**Table 1:** *Bacillus* spore viability after electron beam irradiation, as measured by flow cytometry (*n* = 6).^a^

EB dose (kGy)	Viable counts ± SD	Non viable counts ± SD
0	45,291 ± 768	4,549 ± 774
5.3	41,369 ± 2,574	8,497 ± 2,516
10.4	39,739 ± 1,409*	10,157 ± 1,398*
16.0^b^	31,124 ± 6,659^†^	16,668 ± 4,033^†^
21.7^b^	4,787 ± 1,436^†^	5,187 ± 1,430^†^
Hypochlorous acid^b, c^	33 ± 11^†^	9840 ± 77^†^

^a^Mean ± standard deviation for viable spores identified in Q2 and nonviable spores in Q3 of Figure 4

^b^Less than 50,000 spores available for evaluation

^c^ *n* = 5

*P ≤ 0.05 by the Tukey-Kramer test, as compared to 0 dose (unirradiated control)

^†^ *P* ≤ 0.001 by the Tukey-Kramer test, as compared to 0 dose (unirradiated control)

**Table 2:** Fluorescence intensity of EB-irradiated *Bacillus* spores after SYTO-9 and propidium iodide (PI) uptake.^a^

EB dose (kGy)	SYTO-9± SD	PI ± SD	SYTO-9/PI
0	9441 ± 4280	9365 ± 1854	1.01
5.3	10293 ± 3203	9286 ± 1487	1.11
10.4	5328 ± 3163	8144 ± 2120	0.65^†^
16.0^b^	3703 ± 1656	9144 ± 1347	0.41^†^
21.7^b^	4190 ± 503	9881 ± 946	0.42^†^

^a^Mean ± standard deviation for spores identified in Q2 of Figure 4

^b^Less than 50,000 spores available to evaluate

^†^ *P* ≤ 0.001 by Tukey-Kramer test, as compared to 0 dose (unirradiated control)

**Table 3:** Forward-angle scatter (FSC) and side-angle scatter (SSC) values of EB-irradiated *Bacillus* spores.^a^

EB dose (kGy)	FSC ± SD	SSC ± SD
0	3109 ± 185	26329 ± 529
5.3	2468 ± 109^†^	28103 ± 286^†^
10.4	2453 ± 127^†^	30162 ± 372^†^
16.0^b^	2513 ± 89^†^	31443 ± 1079^†^
21.7^b^	2628 ± 123^†^	31854 ± 922^†^

^a^Mean ± standard deviation for spores identified in Q2 of Figure 4

^b^Less than 50,000 spores available to evaluate

^†^ *P* ≤ 0.001 by Tukey-Kramer test, as compared to 0 dose (unirradiated control).
